# Synergistic chondroprotective effects of curcumin and resveratrol in human articular chondrocytes: inhibition of IL-1β-induced NF-κB-mediated inflammation and apoptosis

**DOI:** 10.1186/ar2850

**Published:** 2009-11-04

**Authors:** Constanze Csaki, Ali Mobasheri, Mehdi Shakibaei

**Affiliations:** 1 Musculoskeletal Research Group, Institute of Anatomy, Ludwig-Maximilians-University Munich, Pettenkoferstrasse 11, 80336 Munich, Germany; 2 Musculoskeletal Research Group, Division of Veterinary Medicine, School of Veterinary Medicine and Science, Faculty of Medicine and Health Sciences, University of Nottingham, Sutton Bonington Campus, Sutton Bonington LE12 5RD, UK

## Abstract

**Introduction:**

Currently available treatments for osteoarthritis (OA) are restricted to nonsteroidal anti-inflammatory drugs, which exhibit numerous side effects and are only temporarily effective. Thus novel, safe and more efficacious anti-inflammatory agents are needed for OA. Naturally occurring polyphenolic compounds, such as curcumin and resveratrol, are potent agents for modulating inflammation. Both compounds mediate their effects by targeting the NF-κB signalling pathway.

**Methods:**

We have recently demonstrated that in chondrocytes resveratrol modulates the NF-κB pathway by inhibiting the proteasome, while curcumin modulates the activation of NF-κB by inhibiting upstream kinases (Akt). However, the combinational effects of these compounds in chondrocytes has not been studied and/or compared with their individual effects. The aim of this study was to investigate the potential synergistic effects of curcumin and resveratrol on IL-1β-stimulated human chondrocytes *in vitro *using immunoblotting and electron microscopy.

**Results:**

Treatment with curcumin and resveratrol suppressed NF-κB-regulated gene products involved in inflammation (cyclooxygenase-2, matrix metalloproteinase (MMP)-3, MMP-9, vascular endothelial growth factor), inhibited apoptosis (Bcl-2, Bcl-xL, and TNF-α receptor-associated factor 1) and prevented activation of caspase-3. IL-1β-induced NF-κB activation was suppressed directly by cocktails of curcumin and resveratrol through inhibition of Iκκ and proteasome activation, inhibition of IκBα phosphorylation and degradation, and inhibition of nuclear translocation of NF-κB. The modulatory effects of curcumin and resveratrol on IL-1β-induced expression of cartilage specific matrix and proinflammatory enzymes were mediated in part by the cartilage-specific transcription factor Sox-9.

**Conclusions:**

We propose that combining these natural compounds may be a useful strategy in OA therapy as compared with separate treatment with each individual compound.

## Introduction

Aging and the proteolytic degradation of extracellular matrix (ECM) macromolecules in articular cartilage in the joint are important catabolic events in osteoarthritis (OA) and rheumatoid arthritis (RA) [1-3]. In OA, synoviocytes and synovial macrophages produce a wide array of inflammatory mediators including prostaglandins, reactive oxygen species and proinflammatory cytokines such as interleukin 1β (IL-1β), interleukin 6 (IL-6) and tumour necrosis factor α (TNF-α). The proinflammatory cytokines in turn stimulate articular chondrocytes and synoviocytes to produce matrix-degrading enzymes such as matrix metalloproteinases (MMPs) and proinflammatory enzymes such as cyclooxygenase-2 (Cox-2). The subsequent release of prostaglandins promotes, sustains and enhances additional cytokine production and inflammation, leading to the destruction and degeneration of the cartilage ECM [4-8]. Several studies have reported that IL-1β and TNF-α are the key proinflammatory cytokines mediating cartilage degradation in patients with RA and OA. IL-1β and TNFα participate in these processes by stimulating chondrocytes and synoviocytes to produce matrix proteases, chemokines, nitric oxide and eicosanoids such as prostaglandins and leukotrienes [5,6,8-10].

Enhanced apoptosis of chondrocytes is now understood to be a sign of progressive cartilage joint degeneration in OA and in rheumatic diseases such as gout [11,12]. IL-1β is well known to induce large-scale apoptosis in chondrocytes in association with mitochondrial dysfunction and depletion of the cellular energy production [13-15]. This process is accompanied by the enhanced synthesis of reactive oxygen species, which in turn, through their interaction with different signal transduction pathways, further stimulate apoptosis [13,16,17], disrupt the mitochondrial membrane potential and ATP production [18], and induce the activation of caspases [19].

Almost all of the proinflammatory factors involved in the pathogenesis and progression of OA and RA are regulated by the transcription factor NF-κB [20]. It is also well known that cellular signalling pathways that involve the Bcl-2/Bax family of proto-oncogenes, the transcription factor NF-κB, TNF-α and IL-1β are able to activate apoptosis [21-26]. These pathways lead to the activation of effector caspases (such as caspase-3), which cleave cellular proteins. During apoptosis, caspases target housekeeping, structural and cytoskeletal proteins and activate inhibitor of caspase-activated deoxyribonuclease or poly(ADP-ribose) polymerase (PARP). The NF-κB subunits and IκBα can also be fragmented by caspases, leading to the repressor form of IκBα [27]. Caspases contribute further to typical morphological features of apoptosis by destruction of the nuclear lamina, which is involved in chromatin organization facilitating heterochromatin condensation at the nuclear envelope. Activation of certain caspases such as caspase-3 plays a pivotal role in initiating apoptosis [28]. Furthermore, it has been demonstrated previously that NF-κB is also involved in part in regulating the master chondrogenic transcription factor Sox-9 [29]. Sox-9 is actively involved in the regulation of cartilage-specific matrix components in chondrocytes such as collagen type II and aggrecan expression, and is thought to play an important role in chondrocyte differentiation [30-33], although other co-factors are also known to be important for collagen type II promotor activation [34,35].

The currently available treatments for OA and RA are only temporarily effective and often result in undesired gastrointestinal side effects. This highlights the need for clinically safe and efficacious new anti-inflammatory agents. Natural compounds, such as curcumin and resveratrol, may circumvent the side effects of nonsteroidal anti-inflammatory drugs and offer new opportunities for OA and RA therapy.

**Figure 1:**
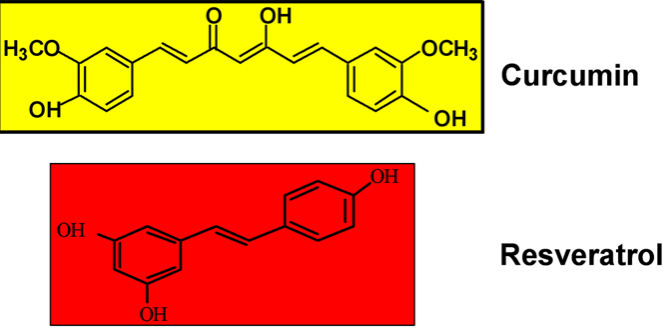
Chemical structures of resveratrol and curcumin. Curcumin is derived from the rhizomes of turmeric (*Curcuma longa*) and resveratrol is found in the skin of red grapes, red berries, peanuts, root extracts of the weed *Polygonum cuspidatum *and numerous other plants. As suggested by their chemical structure, both compounds are polyphenols and therefore they exhibit similar properties as anti-oxidative and anti-inflammatory agents and can act as free radical scavengers.

Curcumin is a potent anti-inflammatory and anti-cancer agent (Figure 1) [36]. Molecular studies have shown that the anti-inflammatory effects of curcumin result from inhibition of the AP-1 and NF-κB pathways: these signalling pathways are activated in response to IL-1β stimulation and activate Cox-2, a key inflammatory mediator involved in downstream activation and release of matrix-degrading MMPs [37-41]. Resveratrol (trans-3,4' -trihydroxystilbene) is a polyphenolic phytoalexin (Figure 1) that demonstrates anti-inflammatory, anti-tumour, immunomodulatory, cardioprotective, anti-oxidative and chemopreventive capabilities [13,42-47]. We recently reported that resveratrol can inhibit IL-1β-induced apoptosis in chondrocytes through downregulation of NF-κB regulated anti-apoptotic gene products mainly through proteasome inhibition [14].

Intracellular signalling is a complex signal communication network, which controls basic biological functions of all cells. Signalling pathways have been found to malfunction in chondrocytes and synovial cells in OA and RA. Effective treatment of arthritic conditions will benefit from a strategy that can simultaneously target multiple cellular signalling pathways to effectively downregulate inflammation in chondrocytes without adverse systemic effects. We have proposed that phytochemicals such as curcumin and resveratrol target the catabolic pathways mediated by the NF-κB signal transduction pathway in cartilage and may be used as clinically safe nutritional factors for the treatment of OA. The aim of the present study was to examine the effects of resveratrol and curcumin, in combination and in isolation on IL-1β-mediated cellular responses and also on the NF-κB signalling transduction pathway, including their potential influence on the master chondrogenic transcription factor Sox-9 and NF-κB-regulated gene products in primary human chondrocytes.

## Materials and methods

### Antibodies

Polyclonal anti-collagen type II, monoclonal anti-β_1_-integrin, and alkaline phosphatase-linked sheep anti-mouse and sheep anti-rabbit secondary antibodies were obtained from Chemicon International (Temecula, CA, USA). Antibodies to β-actin and to ubiquitin were from Sigma (Munich, Germany). Antibodies raised against anti-active caspase-3, MMP-9 and MMP-3 were purchased from R&D Systems (Abingdon, UK). Cyclooxygenase-2 antibody was obtained from Cayman Chemical (Ann Arbor, MI, USA). Antibodies against p65, pan-IκBα, Bcl-2, Bcl-xL and TNF-α receptor-associated factor 1 (TRAF1) were obtained from Santa Cruz Biotechnology (Santa Cruz, CA, USA). Antibodies against phospho-specific IκBα (Ser 32/36) and against anti-phospho-specific p65 (Ser536) were obtained from Cell Technology (Beverly, MA, USA). Anti-IκBα kinase (anti-IKK)-α and anti-IKK-β were obtained from Imgenex (Hamburg, Germany). Anti-vascular endothelial growth factor (anti-VEGF) antibody was purchased from NeoMarkers (Fremont, CA, USA). Monoclonal anti-PARP antibodies were purchased from Becton Dickinson (Heidelberg, Germany). Sox-9 antibody was purchased from Acris Antibodies GmbH (Hiddenhausen, Germany).

All antibodies were used at concentrations and dilutions recommended by the manufacturer (dilutions ranged from 1:100 for immunomorphological experiments to 1:10,000 for western blot analysis).

### Growth medium and chemicals

Growth medium (Ham's F-12/DMEM (50/50) containing 10% FCS, 25 μg/ml ascorbic acid, 50 IU/ml streptomycin, 50 IU/ml penicillin, 2.5 μg/ml amphotericin B, essential amino acids and L-glutamine) was obtained from Seromed (Munich, Germany). Trypsin/ethylenediamine tetraacetic acid (EC 3.4.21.4) was purchased from Sigma. Epon was obtained from Plano (Marburg, Germany). The alkaline phosphatase based APAAP-kit was purchased from Dako (Carpinteria, CA, USA). Resveratrol was purchased from Sigma. Curcumin was purchased from Indsaff (Punjab, India). Resveratrol was prepared as a 100 mg/ml solution in ethanol and then further diluted in cell culture medium. Curcumin was diluted in DMSO as a 5,000 μM concentration and then further diluted in cell culture medium. IL-1β was obtained from Strathman Biotech GmbH (Hannover, Germany).

Peptide aldehydes and the specific proteasome inhibitor *N*-Ac-Leu-Leu-norleucinal (ALLN) were obtained from Boehringer Mannheim (Mannheim, Germany).

### Chondrocyte isolation and culture

Cartilage tissue samples from healthy femoral head articular cartilage obtained during joint replacement surgery for femoral neck fractures were used to isolate primary human articular chondrocytes [48]. Cartilage slices were digested primarily with 1% pronase for 2 hours at 37°C and subsequently with 0.2% (v/v) collagenase for 4 hours at 37°C. Primary chondrocytes were cultured at a density of 200,000 cells in 60 mm petri dishes in monolayer culture for a period of 24 hours at 37°C with 5% carbon dioxide. Cartilage samples were derived from human patients with full informed consent and local ethics committee approval.

### Experimental design

Chondrocyte monolayer cultures were washed three times with serum-starved medium and incubated for 1 hour with serum-starved medium (0.5% FCS). Serum-starved human articular chondrocytes were either left untreated, treated with 10 ng/ml IL-1β alone for the indicated time periods, or pre-treated with 50 μM resveratrol, 50 μM curcumin or 50 μM resveratrol and 50 μM curcumin for 4 hours followed by co-treatment with 10 ng/ml IL-1β and 50 μM resveratrol, or 50 μM curcumin or 50 μM resveratrol and 50 μM curcumin for 24 hours or for the indicated time periods.

### Cell viability and proliferation assay

The effects of resveratrol/curcumin on the cytotoxic effects of IL-1β were examined by the 3-(4,5-dimethylthiazol-2-yl)-2,5-diphenyltetrazolium bromide (MTT kit; Sigma) uptake method as previously described [13]. Briefly, for the cell proliferation assay, 5,000 chondrocytes per well were cultured for 24 hours in a 96-well-plate and then treated with 10 ng/ml IL-1β, 50 μM resveratrol, 50 μM curcumin, 50 μM resveratrol and 50 μM curcumin, or pre-treated with 50 μM resveratrol, 50 μM curcumin, or 50 μM resveratrol and 50 μM curcumin for 4 hours and then co-treated with 10 ng/ml IL-1β, or left untreated and evaluated after 24 hours at 37°C.

For evaluation, the medium was removed and 100 μl fresh medium and 10 μl MTT solution (5 mg/ml PBS, sterile) were added to each well and incubated for 4 hours at 37°C/5% carbon dioxide. Subsequently, 100 μl MTT solubilization solution was added and the plates incubated until the cells were bleached. The transmission signal was determined at 570 nm using a microplate reader (Bio-Rad, Munich, Germany). A sample without cell loading was used as a baseline value. The assay was performed in triplicate and the results are provided as mean values with standard deviations from three independent experiments.

### Poly(ADP-ribose) polymerase cleavage assay

To determine the cleavage products of the DNA repair enzyme PARP, serum-starved chondrocytes were cultured for 24 hours and then treated with 10 ng/ml IL-1β, with 50 μM resveratrol, 50 μM curcumin, and 50 μM resveratrol and 50 μM curcumin, or pre-treated with 50 μM resveratrol, 50 μM curcumin, and 50 μM resveratrol and 50 μM curcumin for 4 hours and then co-treated with 10 ng/ml IL-1β, or left untreated for 24 hours at 37°C. Whole cell extracts were prepared and lysed in lysis buffer (20 mM Tris, pH 7.4, 250 mM NaCl, 2 mM ethylenediamine tetraacetic acid, pH 8.0, 0.1% Triton X-100, 0.01 g/ml aprotinin, 0.005 g/ml leupeptin, 0.4 mM phenylmethylsulfonylfluoride, and 4 mM NaVO_4_). Lysates were spun at 14,000 rpm for 10 minutes to remove insoluble material, resolved by 7.5% SDS-PAGE, and probed with PARP antibodies.

### NF-κB activation assay

The effect of resveratrol/curcumin on the IL-1β-induced nuclear translocation of p65 was examined by an immunocytochemical method (the APAAP method) as described previously [14]. Briefly, chondrocytes seeded on glass plates either were treated with 10 ng/ml IL-1β for 0, 5, 15 and 30 minutes alone, or were pre-treated with resveratrol 50 μM and curcumin 50 μM for 4 hours and then co-treated with 10 ng/ml IL-1β for 0, 5, 15 and 30 minutes. After incubation, cells were fixed for 10 minutes in ice-cold methanol, washed twice (5 minutes) in Tris-buffered saline (TBS) at ambient temperature and then pre-incubated with normal serum for 10 minutes at ambient temperature. The cells were incubated with the primary antibodies (anti-p65) in a humidified chamber overnight at 4°C. Cells were then rinsed twice with (TBS). After washing again, incubation with the dual-system bridge antibodies was performed and cells were treated with the dual-system APAAP complex for 30 minutes at ambient temperature. Cells were thoroughly rinsed with (TBS) and counter-stained with new fuchsin for 30 minutes at ambient temperature. Finally, cells were washed, air dried and mounted in Kaisers' glycerol gelatin prior to examination in an Axiophot 100 light microscope (Zeiss, Jena, Germany).

### Transmission electron microscopy

Samples were fixed for 1 hour with Karnovsky fixative followed by post-fixation in 1% OsO_4 _solution (0.1 M phosphate buffer). Monolayer cell pellets were rinsed and dehydrated in an ascending alcohol series before being embedded in Epon and cut on a Reichert-Jung Ultracut E (Darmsadt, Germany). Ultrathin sections were contrasted with 2% uranyl acetate/lead citrate. A transmission electron microscope (TEM 10; Zeiss) was used to examine the cultures.

### Electron microscopic evaluation of apoptotic cell death

Serum-starved chondrocytes were exposed to 10 ng/ml IL-1β alone for 0, 2, 4 and 8 hours or were pre-stimulated with 50/50 μM resveratrol/curcumin alone for 4 hours and then co-treated with IL-1β (10 ng/ml) for 1, 12, 24 and 48 hours. Ultra-thin sections of the samples were prepared and evaluated with an electron microscope (TEM 10; Zeiss). For statistical analysis, the number of cells with morphological features of apoptotic cell death was determined by scoring 100 cells from 20 different microscopic fields.

### Isolation of chondrocyte nuclei

Cells were trypsinized and washed twice in 1 ml ice-cold PBS. The supernatant was carefully removed. The cell pellet was re-suspended in 400 μl hypotonic lysis buffer containing protease inhibitors and was incubated on ice for 15 minutes. Then 12.5 μl of 10% NP-40 were added and the cell suspension was vigorously mixed for 15 seconds. The extracts were centrifuged for 1.5 minutes. The supernatants (cytoplasmic extracts) were frozen at -70°C. Then 25 μl ice-cold nuclear extraction buffer were added to the pellets and incubated for 30 minutes with intermittent mixing. Extracts were centrifuged and the supernatant (nuclear extracts) transferred to pre-chilled tubes for storage at -70°C.

### Western blot analysis

To determine the effect of resveratrol/curcumin on IL-1β-dependent IκBα phosphorylation, IκBα degradation and p65 translocation, whole cell lysates, cytoplasmic and nuclear extracts of chondrocyte monolayers were prepared and fractioned by SDS-PAGE [14,48,49]. The total protein concentration of whole cell, nuclear and cytoplasmic extracts (30 μg) was determined using the bicinchoninic acid assay system (Uptima; Interchim, Montlucon, France) using BSA as a standard. Equal quantities (500 ng protein per lane) of total proteins were separated by SDS-PAGE (5%, 7.5%, 12% gels) under reducing conditions.

The separated proteins were transferred onto nitrocellulose membranes. Membranes were pre-incubated in blocking buffer (5% (w/v) skimmed milk powder in PBS/0.1% Tween 20) for 1 hour, and were incubated with primary antibodies against p65, IκBα, p-IκBα, VEGF, Cox-2, MMP-3, MMP-9, active caspase-3, PARP, Bcl-2, Bcl-xL, TRAF1, collagen type II, Sox-9 and β-Actin (overnight, 4°C). Membranes were washed three times with blocking buffer, and were incubated with alkaline phosphatase-conjugated secondary antibodies for 30 minutes. They were finally washed three times in 0.1 M Tris, pH 9.5, containing 0.05 M MgCl_2 _and 0.1 M NaCl. Nitroblue tetrazolium and 5-bromo-4-chloro-3-indoylphosphate (*p*-toluidine salt; Pierce, Rockford, IL, USA) were used as substrates to reveal alkaline phosphatase-conjugated specific antigen-antibody complexes. The density (specific binding) of each band was measured by densitometry using Quantity One (Bio-Rad Laboratories Inc., Munich, Germany).

### Immune complex kinase assay

To test the effect of resveratrol or curcumin on IL-1β-induced IKK activation, immune complex kinase assays were performed. The IKK complex was immunoprecipitated from whole cell lysates with antibodies against IKK-α and IKK-β and subsequently incubated with protein A/G-agarose beads (Pierce, Ulm, Germany). After 2 hours of incubation, the beads were washed with lysis buffer and resuspended in a kinase assay solution containing 50 mM HEPES (pH 7.4), 20 mM MgCl_2_, 2 mM dithiothreitol, 10 μM unlabelled ATP and 2 mg substrate GST-IκBα (amino acids 1 to 54), and were incubated at 30°C for 30 minutes. This was followed by boiling in SDS-PAGE sample buffer for 5 minutes. The proteins were transferred to a nitrocellulose membrane after SDS-PAGE under reducing conditions as described above.

Phosphorylation of GST-IκBα was assessed using a specific antibody against phospho-specific IκBα (Ser 32/36). To demonstrate the total amounts of IKK-α and IKK-β in each sample, whole cell lysates were transferred to a nitrocellulose membrane after SDS-PAGE under reducing conditions as described above. Detection of IKK-α and IKK-β was performed by immunoblotting with either anti-IKK-α or anti-IKK-β antibodies.

### Statistical analysis

The results are expressed as the means ± standard deviation of a representative experiment performed in triplicate. The means were compared using Student's *t *test assuming equal variances. *P *< 0.05 was considered statistically significant.

## Results

### Effects of resveratrol and curcumin on human chondrocyte viability and proliferation

**Figure 2:**
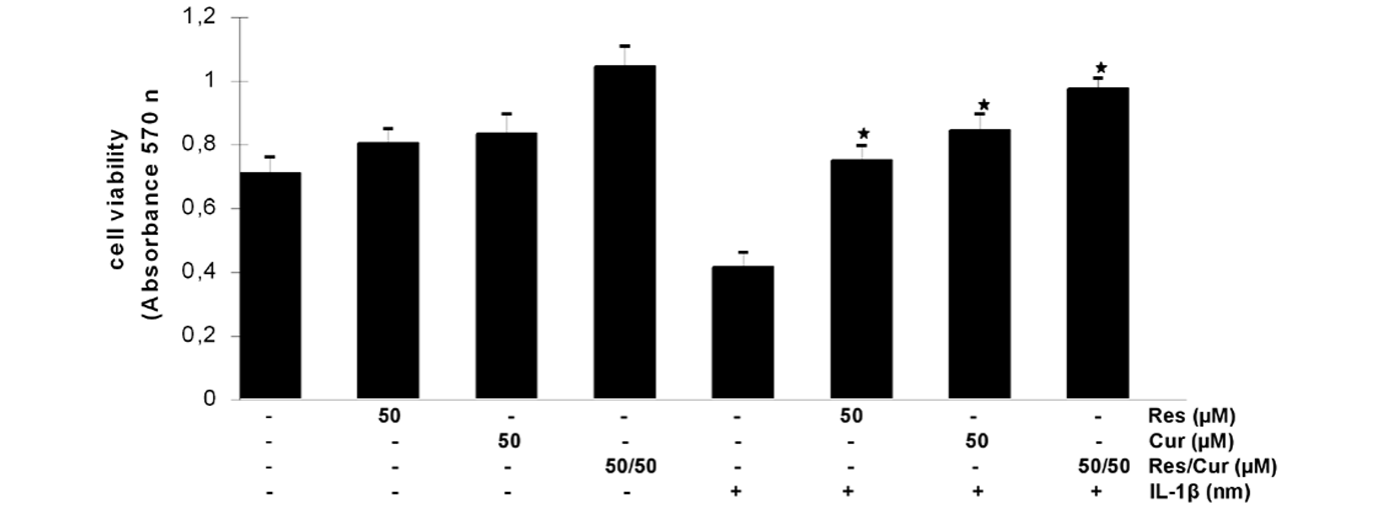
Effects of resveratrol and curcumin and IL-1β on the viability and proliferation of primary chondrocytes *in vitro*. To evaluate the effect of curcumin, resveratrol and/or IL-1β-induced cytotoxicity, primary chondrocytes were treated with 10 ng/ml IL-1β, 50 μM resveratrol, 50 μM curcumin, 50 μM resveratrol and 50 μM curcumin; alternatively they were pre-treated with 50 μM resveratrol, 50 μM curcumin, 50 μM resveratrol and 50 μM curcumin for 4 hours and then co-treated with 10 ng/ml IL-1β, or were left untreated and evaluated after 24 hours using the MTT method. In cells treated with either curcumin, resveratrol or a combination of both, the cytotoxic effects induced by IL-1β were significantly decreased (*) and cell viability was comparable with control cultures.

In previous studies we have demonstrated that IL-1β-induced NF-κB activation is cytotoxic to human chondrocytes [13,14]. In the present study we evaluated the effects of resveratrol and curcumin on this IL-1β-induced cytotoxicity. Proliferation and viability assays performed with the MTT test demonstrated that both resveratrol and curcumin significantly decreased the cytotoxic effects induced by IL-1β (Figure 2). As these data indicated that both phytochemicals have positive and similar properties on human chondrocytes, we investigated the effects of combining resveratrol (50 μM) and curcumin (50 μM) on chondrocyte viability and proliferation. The results showed a positive effect of combining both phytochemicals with regard to cell viability and proliferation on inhibiting the IL-1β-induced cytotoxicity on human chondrocytes (Figure 2).

### Resveratrol and curcumin inhibit IL-1β-induced mitochondrial changes and apoptosis in chondrocytes

Work from our group previously demonstrated that phytochemical agents such as resveratrol and curcumin suppress IL-1β-induced apoptosis in human chondrocytes through inhibition of NF-κB-mediated signalling pathways [13,14]. The objective of the present study was to determine whether curcumin and resveratrol can act synergistically to modulate the cytotoxic effects of IL-1β in human chondrocytes. Primary human chondrocytes were exposed to the indicated concentrations of resveratrol and/or curcumin alone or with IL-1β as described in Materials and methods, and the effect of resveratrol and/or curcumin on IL-1β-induced apoptosis was examined at the ultrastructural level using transmission electron microscopy.

**Figure 3:**
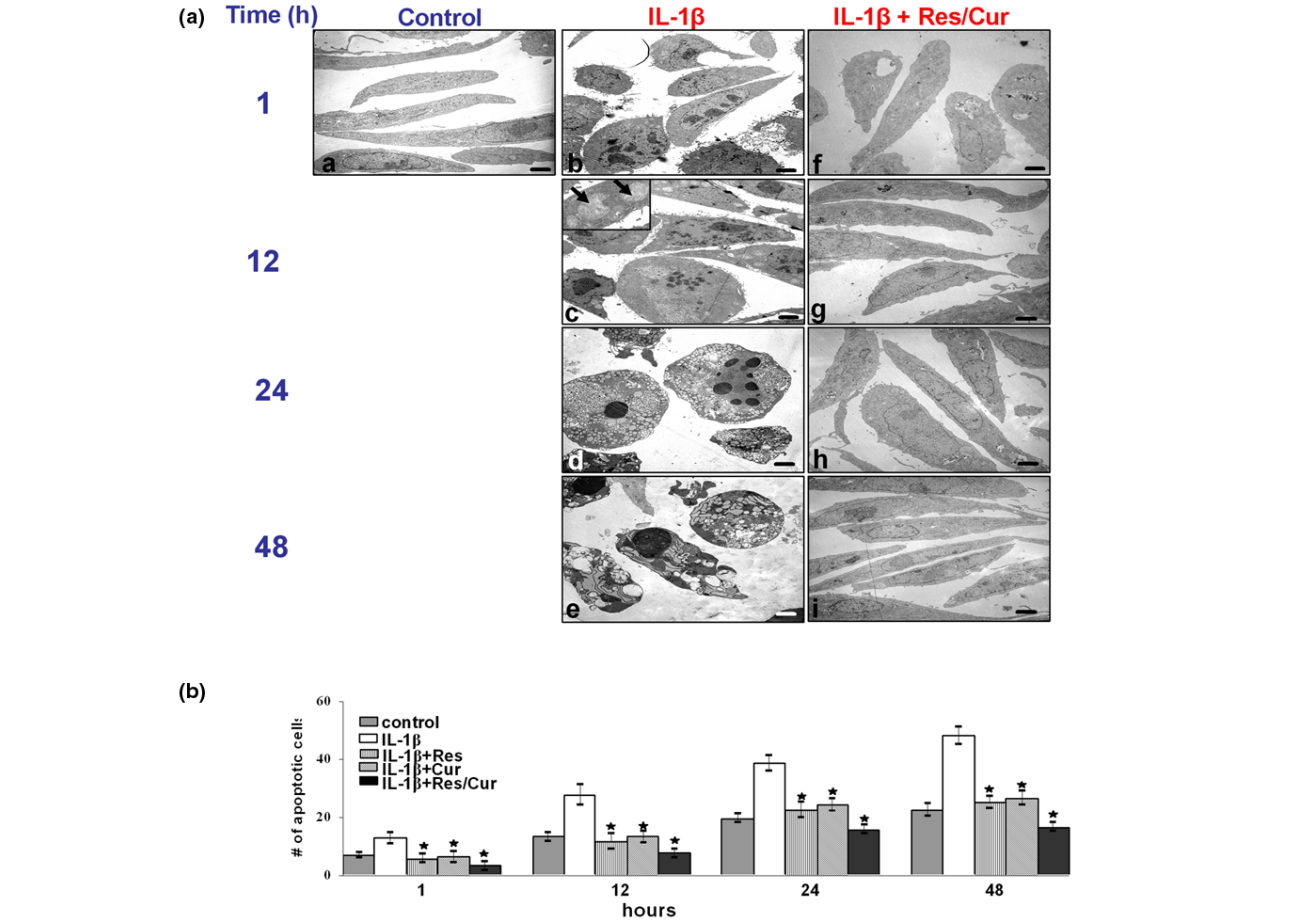
Effects of resveratrol and curcumin on IL-1β-induced mitochondrial changes and apoptosis in primary chondrocytes. **(a) **Transmission electron microscopy was performed to demonstrate the effects of resveratrol and curcumin on IL-1β-stimulated primary chondrocytes in monolayer culture at an ultrastructural level. Untreated control cultures consisted of vital, active chondrocytes containing mitochondria, rough endoplasmic reticulum and many other cell organelles (panel a). In contrast, stimulation of chondrocytes with 10 ng/ml IL-1β for 1, 12, 24, and 48 hours resulted in degenerative changes in the cells. After 1 hour, chondrocytes became rounded and the nucleus contained more condensed chromatin (panel b). After 12 hours, multiple vacuoles, swelling of rough endoplasmic reticulum and clustering of swollen mitochondria were visible (panel c). Inset: arrows demonstrate swollen mitochondria. Longer incubations of 24 to 48 hours led to the formation of apoptotic bodies and cell lysis (panels d to e). Treatment of IL-1β-stimulated primary chondrocytes with resveratrol and curcumin (both at 50 μM), however, inhibited the adverse effects of IL-1β (panels f-i), and after 48 hours of treatment (panel i) chondrocytes demonstrated large, flattened cells with numerous microvilli-like processes, mitochondria and endoplasmic reticulum comparable with control cultures. **(b) **To quantify apoptosis in these cultures, 100 cells from 20 microscopic fields were counted. The number of apoptotic cells was highest in cultures stimulated with IL-1β alone and rose steadily over the entire culture period. In contrast, treatment of IL-1β-stimulated cultures with resveratrol and/or curcumin inhibited the apoptotic effects of IL-1β and the number of apoptotic cells remained significantly lower over the entire culture period (*).

Untreated primary human chondrocytes exhibited a typical rounded or flattened shape with small cytoplasmic processes, a large mostly euchromatic nucleus with nucleoli and a well-structured cytoplasm (Figure 3a, panel a). Treatment of chondrocytes with 10 ng/ml IL-1β for 1, 12, 24 and 48 hours led to degenerative morphological changes (Figure 3a, panels b-e) such as multiple vacuoles, swelling of rough endoplasmic reticulum, clustering of swollen mitochondria (Figure 3a, panel c, inset) and degeneration of other cell organelles. After longer incubation periods (24-48 hours), more severe features of cellular degeneration such as condensed heterochromatin in the cell nuclei and multiple vacuoles were observed. The flattened monolayer chondrocytes became more and more rounded, lost their microvilli-like processes and became apoptotic (Figure 3a, panels c and d). Treatment with either resveratrol or curcumin alone (not shown) or in combination significantly reduced the cytotoxic and apoptotic effects of IL-1β (Figure 3a, panels f-i).

Quantification of apoptosis was achieved by counting the number of apoptotic cells in the samples evaluated by transmission electron microscopy (Figure 3b). In untreated control cultures, the number of cells with apoptotic features in transmission electron microscopy increased with the culture time, as primary chondrocytes started to de-differentiate and degenerate. IL-1β treatment of cultures increased the number of cells with apoptotic features. In contrast, pre-treatment with the phytochemical agents resulted in cells with fewer apoptotic features. We deduce that the lower quantities of apoptotic cells in treated cultures in comparison with control cultures is due to the fact that the phytochemical agents prevent de-differentiation of the primary chondrocytes by stabilizing and stimulating cell metabolism, thus preventing them from becoming apoptotic. This demonstrates that curcumin and resveratrol inhibit the cytotoxic and apoptotic effects induced by IL-1β in chondrocytes (Figure 3a, b).

**Figure 4:**
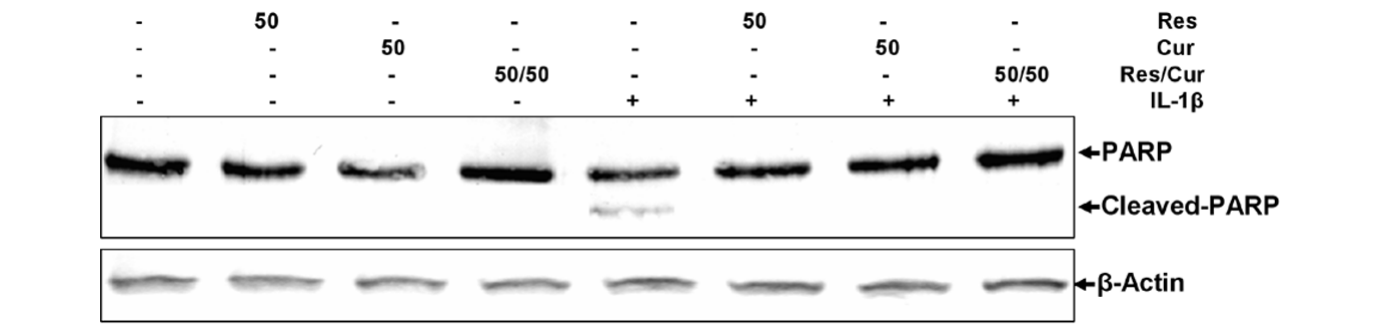
Effects of resveratrol and curcumin on IL-1β-induced apoptosis as demonstrated by poly(ADP-Ribose) polymerase cleavage in primary chondrocytes. As IL-1β-mediated, caspase-induced cleavage of the DNA repair enzyme poly(ADP-Ribose) polymerase (PARP) is a sign of apoptosis, primary chondrocyte cultures were treated with 10 ng/ml IL-1β, 50 μM resveratrol, 50 μM curcumin, 50 μM resveratrol and 50 μM curcumin, or pre-treated with 50 μM resveratrol, 50 μM curcumin, 50 μM resveratrol and 50 μM curcumin for 4 hours and then co-treated with 10 ng/ml IL-1β, or left untreated for 24 hours. Equal amounts (500 ng protein per lane) of total protein were separated by 7.5% SDS-PAGE and analysed by immunoblotting with anti-PARP antibody. Stimulation of chondrocytes with IL-1β alone induced PARP cleavage. Pre-treatment with either resveratrol, curcumin or a combination of both inhibited IL-1β-induced PARP cleavage, however, and levels seen were similar to control cultures. Synthesis of the housekeeping protein β-actin remained unaffected.

Western blot analysis was performed with antibodies against PARP, since cell degeneration and apoptosis is marked by enhanced caspase-mediated cleavage of the DNA repair enzyme PARP (Figure 4). Pre-treatment with either resveratrol, curcumin or the combination of both inhibited IL-1β-induced PARP cleavage, and the levels were similar to control cultures.

Taken together, these results indicate that resveratrol and curcumin synergistically exert anti-apoptotic and anti-cytotoxic effects and counteract IL-1β-induced apoptosis in human chondrocytes.

### Resveratrol and curcumin stimulate the expression of anti-apoptotic and inhibit pro-apoptotic gene products in chondrocytes

**Figure 5:**
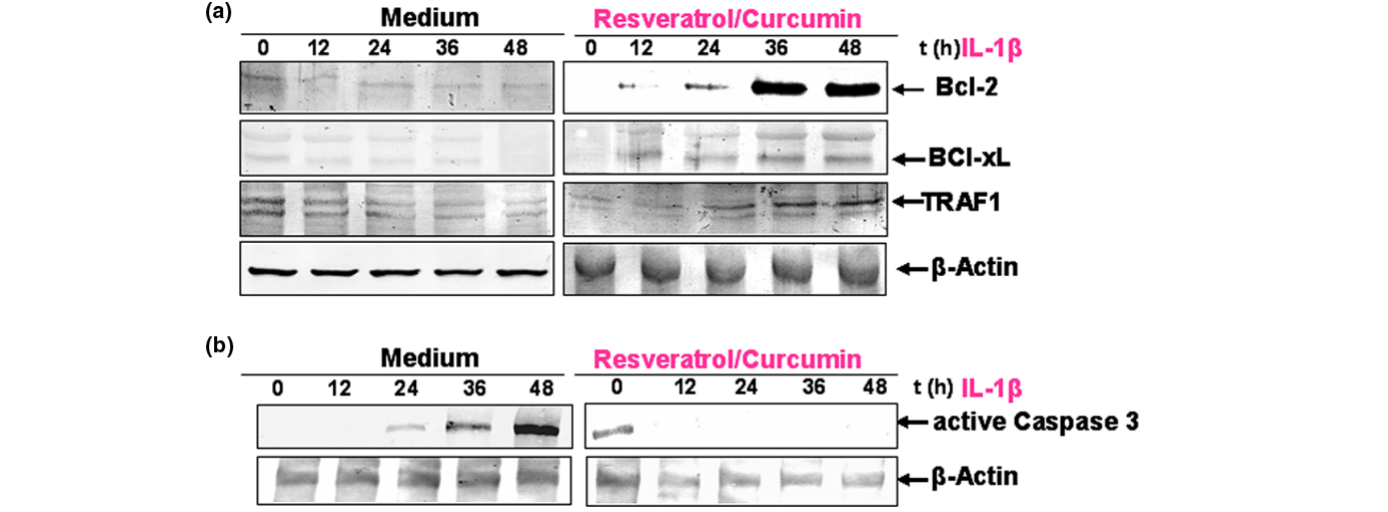
Effects of resveratrol and curcumin on IL-1β-induced NF-κB-dependent gene products in primary chondrocytes. **(a) **The effect of resveratrol/curcumin on IL-1β-induced NF-κB-dependent anti-apoptotic gene products in primary chondrocytes was studied. To determine whether resveratrol and curcumin treatment actively stimulates the production of anti-apoptotic gene products, primary chondrocyte cultures were either stimulated for 0, 12, 24, and 48 hours with 10 ng/ml IL-1β or pre-treated with resveratrol and curcumin (50/50 μM) followed by 0, 12, 24, and 48 hours stimulation with 10 ng/ml IL-1β. Equal amounts (500 ng protein per lane) of total proteins were separated by 10% SDS-PAGE and analysed by immunoblotting with anti-Bcl-2, anti-Bcl-xL and anti-TNF-α receptor-associated factor 1 (anti-TRAF1) antibodies. A time-dependent downregulation of the expression of Bcl-2, Bcl-xL and TRAF1 by IL-1β was observed. In contrast, pre-treatment with resveratrol and curcumin resulted in a time-dependent increase of these anti-apoptotic proteins. Synthesis of the housekeeping protein β-actin remained unaffected. **(b) **The effect of resveratrol/curcumin on IL-1β-induced NF-κB-dependent pro-apoptotic protein caspase-3 was also studied in primary chondrocytes. Whole cell lysates of primary chondrocyte cultures were either stimulated for 0, 12, 24, and 48 hours with 10 ng/ml IL-1β or pre-treated with resveratrol and curcumin (50/50 μM) followed by 0, 12, 24, and 48 hours of stimulation with 10 ng/ml IL-1β-, and evaluated with western blot analysis to examine the effect on the pro-apoptotic protein caspase-3. Equal amounts (500 ng protein per lane) of total proteins were separated by 12% SDS-PAGE and analysed by immunoblotting with an antibody against active caspase-3. Stimulation of the cultures with IL-1β resulted in a time-dependent activation of caspase-3. In contrast, combinational treatment of resveratrol and curcumin inhibited caspase-3 activation in a time-dependent manner. Synthesis of the housekeeping protein β-actin was not affected.

It is known that NF-κB regulates the expression of the anti-apoptotic proteins Bcl-2, Bcl-xL and TRAF1 [50,51]. To evaluate whether resveratrol and curcumin can modulate the expression of these anti-apoptotic genes products, we examined IL-1β-stimulated primary human chondrocytes with or without pre-treatment of resveratrol and curcumin by western blot analysis (Figure 5a). IL-1β inhibited the expression of Bcl-2, Bcl-xL and TRAF1 in a time-dependent manner. In contrast to this, the combinational treatment of resveratrol and curcumin stimulated the expression of the above-mentioned anti-apoptotic proteins in the same manner in chondrocytes (Figure 5a).

Furthermore, we wanted to know whether resveratrol and curcumin also suppress the IL-1β-induced pro-apoptotic gene product, activated caspase-3, in the same cell cultures. To determine this, primary human chondrocytes were incubated with IL-1β (10 ng/ml) alone for the indicated time or were pre-incubated with resveratrol and curcumin (50/50 μM) for 4 hours and then co-treated with IL-1β (10 ng/ml) for the indicated time. As shown in Figure 5b, pre-treatment with resveratrol and curcumin significantly downregulated the level of biologically active caspase-3 in IL-1β-stimulated cultures compared with primary human chondrocytes stimulated with IL-1β alone.

### Resveratrol and curcumin inhibit IL-1β-induced NF-κB-dependent proinflammatory and matrix degradation gene products in chondrocytes

**Figure 6:**
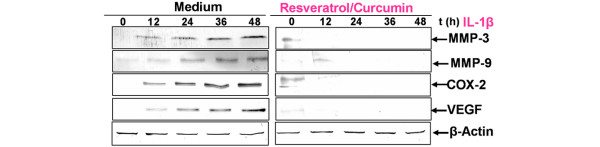
Effects of resveratrol and curcumin on IL-1β-induced NF-κB-dependent proinflammatory and matrix-degrading gene products in primary chondrocytes. To evaluate whether resveratrol and curcumin exert time-dependent effects on IL-1β-induced NF-κB-dependent expression of proinflammatory and matrix-degrading gene products, primary chondrocyte cultures were either stimulated for 0, 12, 24, and 48 hours with 10 ng/ml IL-1β or pre-treated with resveratrol and curcumin (50 μM each) followed by 0, 12, 24, and 48 hours of stimulation with 10 ng/ml IL-1β; after extraction of whole cell lysates (500 ng protein per lane), they were probed for the expression of matrix metalloproteinase (MMP)-3, MMP-9, cylcooxygenase-2 (Cox-2) and vascular endothelial growth factor (VEGF) by western blot analysis. Stimulation of IL-1β alone consistently resulted in time-dependent production of MMP-3, MMP-9, Cox-2 and VEGF. In contrast, pre-treatment with resveratrol and curcumin downregulated MMP-3, MMP-9, Cox-2 and VEGF time dependently. Synthesis of the housekeeping protein β-actin was unaffected.

We investigated whether resveratrol and curcumin can modulate IL-1β-induced NF-κB-regulated gene products involved in the inflammation and degradation processes in cartilage tissue. It has been shown previously in chondrocytes that IL-1β stimulation activates Cox-2, VEGF, MMP-3 and MMP-9 expression. We therefore investigated whether both natural products are able to inhibit the IL-1β-induced expression of these proteins. Primary human chondrocytes with or without pre-treatment with resveratrol and curcumin were examined for IL-1β-induced gene products by western blot analysis using specific antibodies (Figure 6). IL-1β induced the expression of Cox-2, MMP-3, MMP-9 and VEGF in a time-dependent manner, and the combinational treatment of resveratrol and curcumin inhibited the expression of the above-mentioned proteins in primary chondrocytes (Figure 6). Synthesis of the housekeeping protein β-actin remained unaffected (Figure 6).

### Effect of resveratrol and/or curcumin on IL-1β-induced inhibition of collagen type II production in chondrocytes

**Figure 7:**
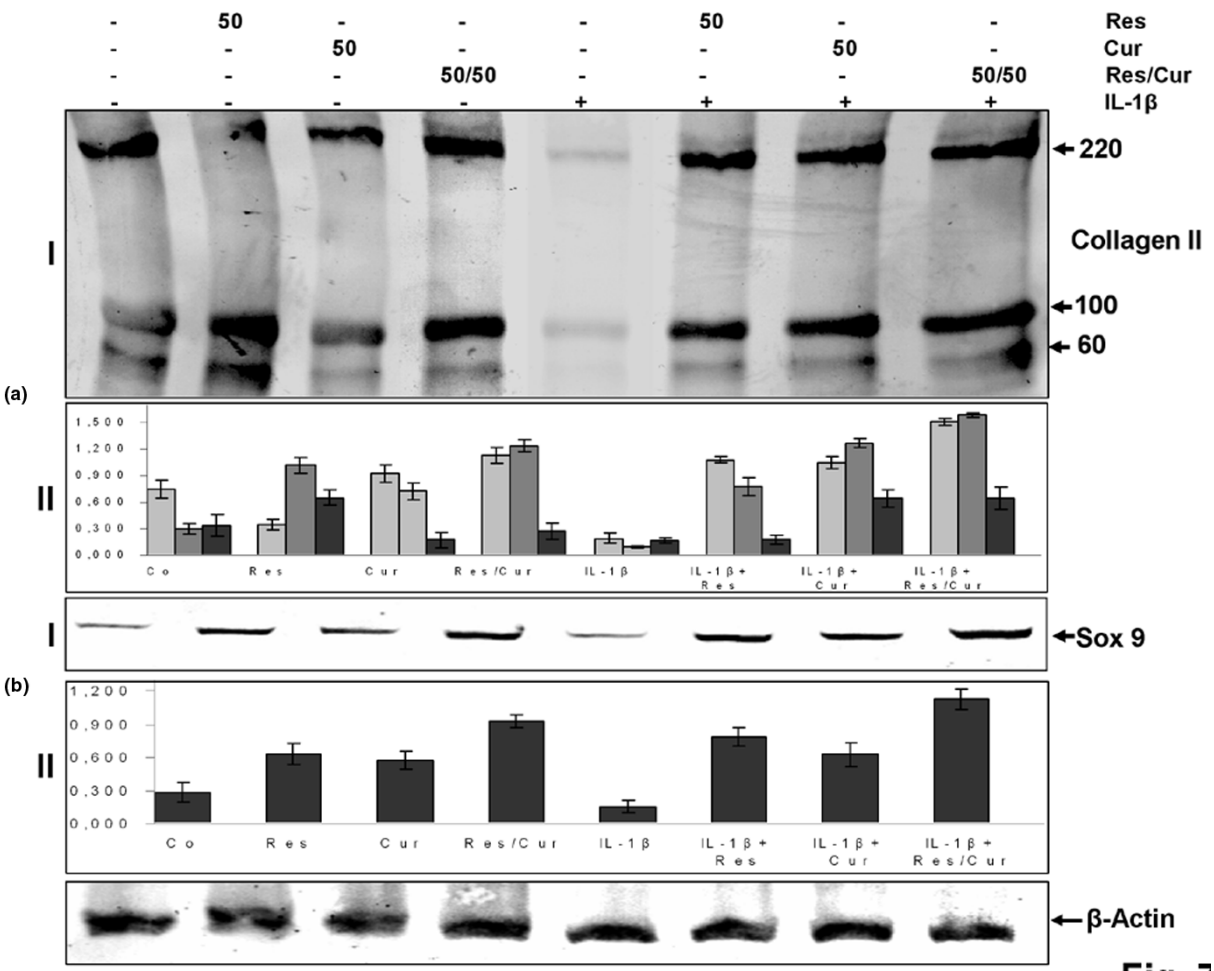
Effects of resveratrol and curcumin on IL-1β-induced inhibition of collagen type II and Sox-9 production in chondrocytes. To evaluate the effects of resveratrol and curcumin on IL-1β-stimulated chondrogenic inhibition in primary chondrocytes, whole cell lysates (500 ng protein per lane) were probed with antibodies to **(a) **collagen type II (panel I), as the most abundant cartilage-specific extracellular matrix protein, and **(b) **the chondrogenic-specific transcription factor Sox-9 (panel I). Cultures were treated with 10 ng/ml IL-1β, 50 μM resveratrol, 50 μM curcumin, 50 μM resveratrol and 50 μM curcumin, or were pre-treated with 50 μM resveratrol, 50 μM curcumin, 50 μM resveratrol and 50 μM curcumin for 4 hours and then co-treated with 10 ng/ml IL-1β, or left untreated for 24 hours. Untreated cultures had strong (a) collagen type II and (b) Sox-9 and stimulation with IL-1β alone greatly reduced collagen type II as well as Sox-9 production. However, pre-treatment of the cultures with resveratrol, curcumin or a combination of both inhibited the adverse effects of IL-1β and the chondrocytes produced large quantities of collagen type II and Sox-9 at levels similar to control cultures. This was confirmed by quantitative densitometry (a, panel II and b, panel II). The mean values and standard deviations from three independent experiments are shown. White, grey and solid bars represent different molecular forms of collagen type II. Synthesis of the housekeeping protein β-actin remained unaffected.

Serum-starved human articular chondrocytes were cultured for 24 hours and then treated with 10 ng/ml IL-1β, 50 μM resveratrol, 50 μM curcumin, and with 50 μM resveratrol and 50 μM curcumin, or were pre-treated with 50 μM resveratrol, 50 μM curcumin, and 50 μM resveratrol and 50 μM curcumin for 4 hours and then co-treated with 10 ng/ml IL-1β, or left untreated and evaluated after 24 hours (Figure 7). Treatment of chondrocytes with 50 μM curcumin, with 50 μM resveratrol or with 50 μM resveratrol and 50 μM curcumin resulted in a stimulation of collagen type II production. Primary human chondrocytes stimulated with IL-1β alone showed a significant downregulation of synthesis of collagen type II. In contrast, pre-treatment of chondrocytes with the phytochemical agents followed by stimulation with IL-1β resulted in an inhibition of cytokine-induced effects on collagen type II production (Figure 7a, panel I). Interestingly, co-treatment of the chondrocytes with combinations of the two phytochemical agents increased the levels of these proteins more than each agent by itself.

### Effect of resveratrol and/or curcumin on Sox-9 in the chondrocyte nucleus

Sox-9 is a master specific transcription factor that controls the expression of chondrocyte-specific ECM protein genes and plays a pivotal role in chondrocyte differentiation [52]. To test the hypothesis that phytochemicals are able to activate the transcription factor Sox-9 in human chondrocytes, monolayer cultures of human chondrocytes were either left unstimulated or stimulated with resveratrol and/or curcumin or were pre-treated with resveratrol and/or curcumin (50/50 μM) for 4 hours and then stimulated with IL-1β for 24 hours, and the cell lysates were analysed by immunoblotting.

The results demonstrated that resveratrol and/or curcumin stimulated Sox-9 expression and inhibited the IL-1β-induced decreased Sox-9 expression (Figure 7b, panel I). Because these data indicate that both phytochemicals have similar properties, we further investigated the cumulative role of resveratrol (50 μM) and curcumin (50 μM) on IL-1β-induced inhibition of Sox-9 expression in chondrocytes. The results suggest that signalling from exposure to extracellular resveratrol and curcumin converge to influence the activity of transcription factors such as Sox-9, which are necessary for the expression of cartilage matrix genes (Figure 7b). Quantitative analysis (Figure 7a, panel II and 7b, panel II) of the western blot results confirmed that resveratrol and/or curcumin increase the expression of collagen type II (Figure 7a, panel II) and Sox-9 (Figure 7b, panel II) and inhibit the IL-1β-induced decrease in collagen type II and Sox-9 expression. Data shown are representative of three independent experiments.

### Resveratrol and curcumin block IL-1β-induced nuclear translocation of NF-κB as revealed by APAAP

NF-κB is an important transcriptional regulator of inflammatory cytokines gene expression and plays a crucial role in inflammatory responses. After phosphorylation, ubiquitination and degradation of IκBα, the NF-κB fragment is translocated to the nucleus where it binds and activates the promoter of target genes. This translocation of NF-κB to the nucleus is necessary for regulation of gene expression by NF-κB [20].

**Figure 8:**
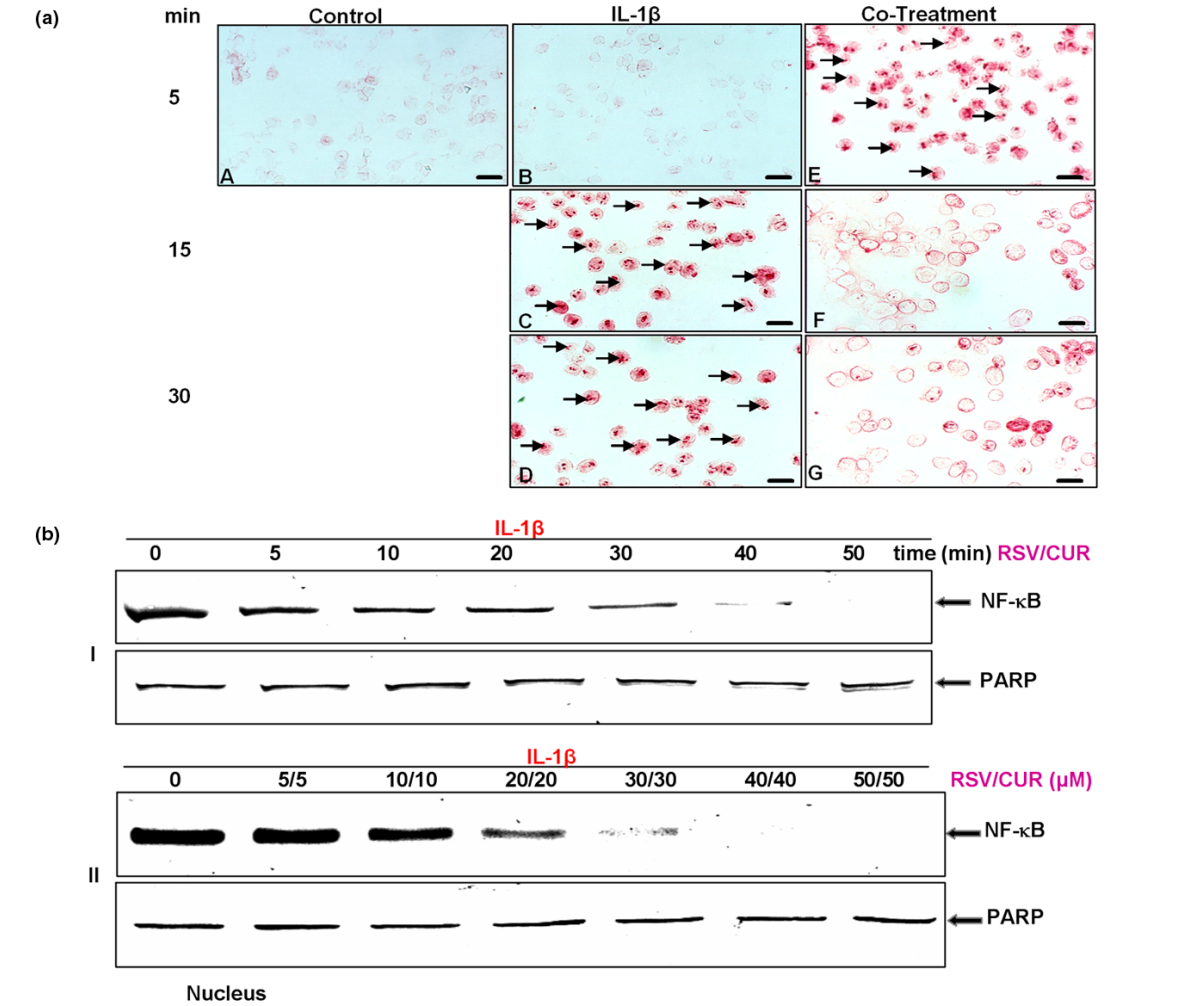
Inhibition of IL-1β-induced NF-κB activation and nuclear translocation by resveratrol and curcumin in primary chondrocytes using APAAP. **(a) **Human chondrocyte cultures either served as controls (panel A, not treated) or treated either with 10 ng/ml IL-1β 5, 15 and 30 minutes alone or pre-treated with 50 μM resveratrol and 50 μM curcumin for 4 hours and then co-treated with 10 ng/ml IL-1β for 5, 15 and 30 minutes, before immunolabelling with phospho-p65 antibodies. In control cells, anti-phospho-p65 labelling was restricted to the cytoplasm (panel A). Cells treated with IL-1β alone revealed nuclear translocation of phospho-p65 (panels B-D) that was inhibited by co-treatment with resveratrol and curcumin (panels E-G). Data shown are representative of three independent experiments. A-G: × 160, bar = 50 μm. **(b) **Panel I: western blot analysis with IL-1β-treated chondrocyte nuclear extracts. Serum-starved chondrocytes were pre-incubated with 50 μM resveratrol and 50 μM curcumin for 5, 10, 20, 30, 40 and 50 minutes, co-treated with 10 ng/ml IL-1β for 30 minutes, and then probed for phospho-p65 by western blot analysis using antibodies to phospho-specific p65 and poly(ADP-Ribose) polymerase (PARP) (control). Resveratrol and curcumin pre-treatment inhibited IL-1β-induced NF-κB activation in a time-dependent manner. NF-κB nuclear translocation was inhibited completely after 50 minutes of pre-treatment with resveratrol and curcumin. Panel II: serum-starved human chondrocytes were pre-incubated with resveratrol and curcumin at various concentrations (5 μM, 10 μM, 20 μM, 30 μM, 40 μM and 50 μM each) for 4 hours followed by 10 ng/ml IL-1β stimulation for 30 minutes. The nuclear extracts (500 ng protein per lane) were probed for phospho-p65 by western blot analysis using antibodies to phospho-specific p65 and PARP (control). A concentration-dependent inhibition of NF-κB nuclear translocation was observed. At a concentration of 40 μM resveratrol and 40 μM curcumin, NF-κB nuclear translocation was completely inhibited. The inhibition of NF-κB nuclear translocation by resveratrol and curcumin is therefore concentration as well as time dependent. Synthesis of PARP remained unaffected in nuclear extracts.

Primary human chondrocytes were either left untreated (Figure 8a, A), or treated with 10 ng/ml IL-1β alone for 5, 15 and 30 minutes (Figure 8a, panels B-D), or pre-treated with resveratrol and/or curcumin (50/50 μM) for 4 hours and then stimulated with IL-1β for the same time periods (Figure 8a, panels E-G). In untreated control cultures, only cytoplasmic labelling of NF-κB was observed (Figure 8a, panel A). After 15 minutes of treatment, IL-1β-stimulated chondrocytes showed a clear and positive labelling for activated NF-κB in the nuclei and to a lesser extent in the cytoplasm of chondrocytes (Figure 8a, panels B-D). Chondrocytes that were pre-treated with resveratrol and curcumin 50/50 μM (4 hours) and then co-treated with IL-1β and resveratrol and curcumin showed positive staining in the cytoplasm and showed a clearly decreased, nuclear NF-κB staining (Figure 8a, panels E-G).

### Resveratrol and curcumin inhibit NF-κB activation caused by IL-1β in a concentration-dependent and time-dependent manner in chondrocytes

To examine whether resveratrol and curcumin block the IL-1β-induced activation of NF-κB, nuclear protein extracts from serum-starved chondrocytes were probed for the phosphorylated form of the p65 NF-κB subunit after pre-treatment with 50 μM resveratrol and 50 μM curcumin for the indicated times followed by 10 ng/ml IL-1β stimulation for 30 minutes (Figure 8b, panel I). Furthermore, chondrocytes were pre-incubated with the indicated concentrations of resveratrol and curcumin for 4 hours followed by co-treatment with 10 ng/ml IL-1β and resveratrol and curcumin for 30 minutes (Figure 8b, panel II). The western blot results confirmed that co-treatment of resveratrol and curcumin had no effect on NF-κB activation. Resveratrol and curcumin, however, inhibited IL-1β-induced NF-κB activation in a time-dependent (Figure 8b, panel I) and a concentration-dependent (Figure 8b, panel II) manner.

### Resveratrol but not curcumin inhibits IL-1β-induced IκBα degradation

Resveratrol and curcumin inhibited IL-1β-induced activation of NF-κB and its translocation to the chondrocyte nucleus. We therefore examined the upstream mechanisms of NF-κB activation by IL-1β in chondrocytes. It is well known that an important pre-requisite for the activation of NF-κB is the phosphorylation and degradation of IκBα, the natural blocker of NF-κB [53,54].

**Figure 9:**
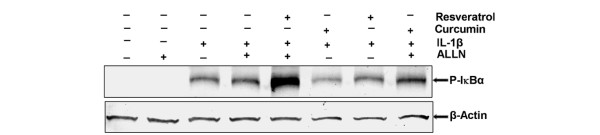
Effects of resveratrol and curcumin treatment on IL-1β-induced IκBα degradation. Serum-starved human chondrocytes were pre-treated with 10 ng/ml IL-1β alone for 4 hours, or with 100 μM *N*-Ac-Leu-Leu-norleucinal (ALLN) for 30 minutes, or pre-treated with 50 μM resveratrol, 50 μM curcumin or 100 μM ALLN alone for 4 hours and then co-treated with IL-1β (10 ng/ml) for 8 hours. Other serum-starved chondrocytes were pre-treated with 50 μM resveratrol or 50 μM curcumin alone for 4 hours and then co-treated with IL-1β (10 ng/ml) for 8 hours. Some cultures were left untreated and evaluated after 12 hours. Cytoplasmic extracts (500 ng protein per lane) were fractionated and then subjected to western blotting with phosphospecific IκBα antibody. The data demonstrate that resveratrol (but not curcumin) inhibits IL-1β-induced IκBα degradation. Data shown are representative of three independent experiments. The same membrane was re-blotted with antibodies to β-actin.

To test whether inhibition of IL-1β-induced NF-κB activation occurs through inhibition of IκBα degradation or through inhibition of IKK activation, we treated chondrocyte cultures for 8 hours with 10 ng/ml IL-1β alone or with 100 μM of the specific proteasome inhibitor ALLN [55], which prevents the degradation of phosphorylated IκBα by the 26S proteasome. Other serum-starved human articular chondrocytes were pre-stimulated with 50 μM resveratrol, 50 μM curcumin or 100 μM ALLN alone for 4 hours and then co-treated with IL-1β (10 ng/ml) for 8 hours. Additionally, other serum-starved human articular chondrocytes were pre-stimulated with 50 μM resveratrol or 50 μM curcumin alone for 4 hours and then co-treated with IL-1β (10 ng/ml) for 8 hours. Some cultures were left untreated and evaluated after 12 hours. The activation of pIκBα in the cytoplasm of the chondrocytes was determined by western blot analysis using anti-IκBα and anti-β-actin (control) antibodies. IL-1β induced IκBα degradation in untreated cultures, but IL-1β could not induce IκBα degradation in resveratrol pre-treated chondrocytes - in contrast to curcumin pre-treated cells (Figure 9). Taken together, these results suggest that in contrast to curcumin resveratrol blocks IL-1β-induced IκBα degradation. Data shown are representative of three independent experiments.

### Resveratrol but not curcumin inhibits IL-1β-dependent ubiquitination of IκBα

**Figure 10:**
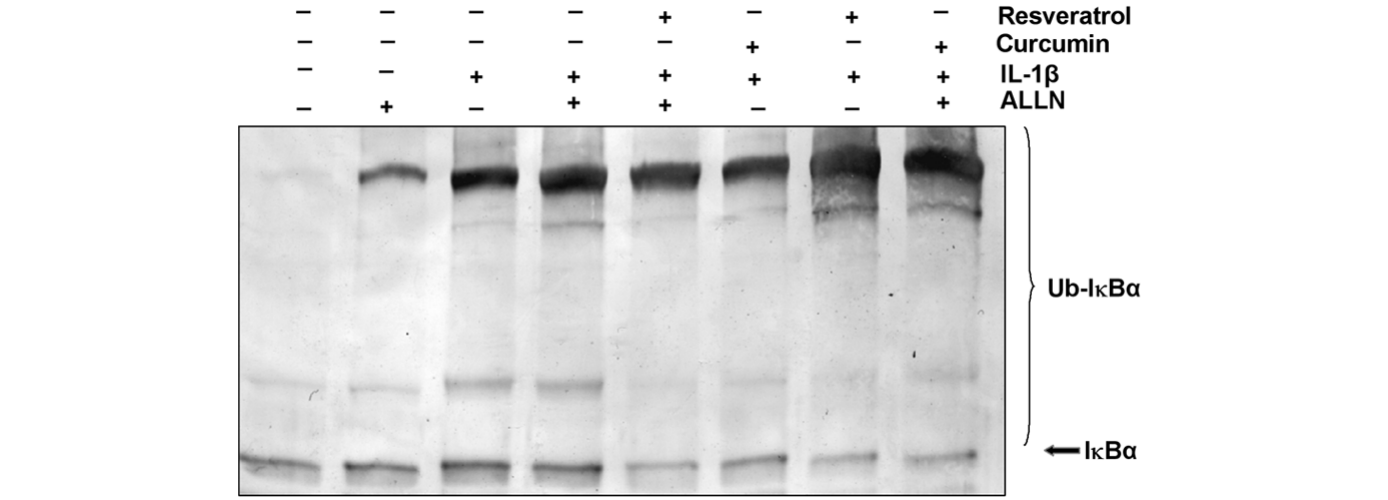
Effects of resveratrol and curcumin treatment on IL-1β-induced IκBα ubiquitination. Serum-starved human chondrocytes were pre-treated with 10 ng/ml IL-1β alone for 4 hours, or 100 μM *N*-Ac-Leu-Leu-norleucinal (ALLN) for 30 minutes, or pre-treated with 50 μM resveratrol, 50 μM curcumin or 100 μM ALLN alone for 4 hours and then co-treated with IL-1β (10 ng/ml) for 8 hours. Other serum-starved chondrocytes were pre-treated with 50 μM resveratrol or 50 μM curcumin alone for 4 hours and then co-treated with IL-1β (10 ng/ml) for 8 hours. Some cultures were left untreated and evaluated after 12 hours. Cytoplasmic extracts were immunoprecipitated with an antibody against IκBα and subjected to western blot analysis using a monoclonal anti-ubiquitin antibody. Resveratrol (but not curcumin), stabilized IL-1β-induced ubiquitination of IκBα. Data shown are representative of three independent experiments.

Next we determined whether resveratrol or curcumin affected the IL-1β-induced IκBα ubiquitination that leads to IκBα degradation. We treated some chondrocyte cultures with 10 ng/ml IL-1β alone or with 100 μM ALLN for 8 hours. Other serum-starved human articular chondrocytes were pre-stimulated with 50 μM resveratrol, 50 μM curcumin or 100 μM ALLN alone for 4 hours and then co-treated with IL-1β (10 ng/ml) for 8 hours. Other serum-starved human articular chondrocytes were pre-stimulated with 50 μM resveratrol or 50 μM curcumin alone for 4 hours and then co-treated with IL-1β (10 ng/ml) for 8 hours. Some cultures were left untreated and evaluated after 12 hours. Western blot analysis using an antibody that detects IκBα indicated that IL-1β induced IκBα ubiquitination, as indicated by high molecular weight bands, and that mainly resveratrol, but not curcumin, suppressed this ubiquitination (Figure 10). Mainly resveratrol, but not curcumin, therefore inhibited IL-1β-induced NF-κB activation by inhibiting phosphorylation, ubiquitination, and degradation of IκBα (Figure 10). Data shown are representative of three independent experiments.

### Resveratrol does not inhibit IL-1β-induced IKK activation

**Figure 11:**
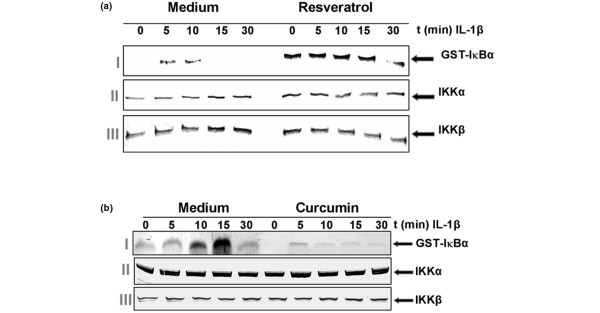
Effects of resveratrol and curcumin treatment on IL-1β-induced IκB kinase activation. Serum-starved primary human chondrocytes were pre-treated with **(a) **50 μM resveratrol or **(b) **50 μM curcumin for 4 hours and then co-treated with IL-1β (10 ng/ml) for the indicated times. Whole cell extracts were immunoprecipitated with an antibody against IκB kinase (IKK)-α and analysed by an immune complex kinase assay. To examine the effect of curcumin on the expression level of IKK proteins, whole cell extracts (500 ng protein per lane) were fractionated by SDS-PAGE and examined using western blot analysis with anti-IKK-α and anti-IKK-β antibodies. Data shown are representative of three independent experiments. The results demonstrate that resveratrol does not affect IL-1β-induced IKK activation.

As we could demonstrate, resveratrol inhibits the degradation and ubiquitination of IκBα. We now further evaluated the effect of resveratrol on IL-1β-induced IKK activation, which is required for IL-1β-induced phosphorylation of IκBα. The results from the immune complex kinase assay showed that IL-1β activated IKK as early as 5 minutes after IL-1β treatment, but that resveratrol did not inhibit IL-1β-induced activation of IKK (Figure 11a, panel I). IL-1β or resveratrol had no direct effect on the expression of IKK protein (Figure 11a, panels II and III).

### Curcumin but not resveratrol inhibits IL-1β-induced IκBα kinase activation

Since we could demonstrate that curcumin inhibits the phosphorylation of IκBα, we then evaluated whether curcumin has an effect on IL-1β-induced IKK activation, which is required for IL-1β-induced phosphorylation of IκBα. Curcumin completely suppressed IL-1β-induced activation of IKK (Figure 11b, panel I). IL-1β or curcumin had no direct effect on the expression of IKK protein (Figure 11b, panels II and III).

## Discussion

The aim of the present study was to determine if the anti-inflammatory and anti-apoptotic effects of resveratrol and curcumin in primary human chondrocytes are mediated by similar signalling mechanisms and whether combining these natural compounds has a synergistic effect on IL-1β-mediated cellular responses, NF-κB-mediated signal transduction pathways and regulation of NF-κB-regulated gene expression.

The study leads to the following findings: IL-1β-induced suppression of chondrocytes viability and proliferation is revoked by resveratrol and curcumin pre-treatment. Stimulation of chondrocytes with IL-1β results in morphological alterations (that is, swollen mitochondria, dilated endoplasmic reticulum and apoptosis) that were abolished through pre-treatment with resveratrol and curcumin. Co-treatment of the IL-1β-stimulated cells with both resveratrol and/or curcumin inhibits activation of PARP cleavage. Resveratrol potentiates the anti-inflammatory and anti-apoptotic effects of curcumin on IL-1β-stimulated chondrocytes, and this correlates with downregulation of NF-κB-specific gene products that are known to mediate inflammation, degradation and apoptosis of chondrocytes in OA. Additionally, both resveratrol and/or curcumin suppressed IL-1β-induced downregulation of the cartilage-specific ECM component collagen type II and of the cartilage-specific master transcription factor Sox-9. The activation and translocation of p65 from the cytoplasm to the nucleus could be inhibited clearly by resveratrol and curcumin in IL-1β-stimulated human chondrocytes. Both resveratrol and curcumin inhibited NF-κB activation in a concentration-dependent and time-dependent manner. Finally, inhibition of NF-κB activation by resveratrol occurred mainly through the accumulation of phosphorylated IκBα, ubiquitinated IκBα and inhibition of proteasome activity - in contrast to this, in the case of curcumin it was mainly caused through inhibition of IKK activation.

Proinflammatory cytokines such as TNF-α and IL-1β have been shown to mediate cartilage degradation and apoptosis in chondrocytes in degenerative joint diseases such as RA and OA in humans as well as in animals. Indeed, cytokine-mediated apoptosis of chondrocytes is believed to play a key role in the pathogenesis of OA [56-59]. These proinflammatory cytokines are produced by activated synoviocytes, macrophages and chondrocytes [60]. They are well known to activate the ubiquitous transcription factor NF-κB, which leads to further production and upregulation of proinflammatory cytokines and enzymes such as Cox-2 and MMPs, which in turn produce prostaglandins and degrade ECM macromolecules leading to cartilage degradation and further joint inflammation [40]. Although numerous effects have been described for resveratrol and curcumin, however, the mechanisms responsible for their anti-inflammatory effects in chondrocytes are not yet clear.

Activation of NF-κB provides the potential link between inflammation and hyperplasia during OA and RA in the joint [61]. The use of a specific NF-κB inhibitor has been reported to result in a significant decrease in joint swelling in mice with collagen-induced arthritis [62]. NF-κB therefore represents an important target for therapeutic strategies aimed at the prophylactic treatment of inflammatory disorders, such as OA and RA.

Resveratrol and curcumin are anti-inflammatory dietary phytochemicals that have previously been shown to antagonize some catabolic effects of TNFα and IL-1β via inhibition of NF-κB in different cell types [13,63-67]. We have previously demonstrated that in chondrocytes resveratrol inhibits NF-κB through suppression of the proteasome activity [14], and this leads to the accumulation of phosphorylated IκBα and inhibition of p65. Curcumin has been shown to inhibit NF-κB activation in chondrocytes [40] and other cell lines of various origins [68]. However, whether curcumin can also inhibit NF-κB activation through inhibition of the proteasome activity or IKK activation in chondrocytes has not been previously reported.

We found that inhibition of IKK by curcumin, which is needed for NF-κB activation, led to inhibition of phosphorylation of both IκBα and p65 but the proteasome was not affected by curcumin. We also found that resveratrol and curcumin in combination stimulated several genes that are regulated by NF-κB, including anti-apoptotic gene products (Bcl-2, Bcl-xL and TRAF1). Resveratrol and curcumin inhibited pro-apoptotic proteins (caspase-3, PARP) and matrix degrading gene products (MMP3 and MMP-9), and angiogenesis and inflammation gene products (VEGF and Cox-2). It has been reported that the expression of Bcl-2 and Bcl-xL is known to be regulated by NF-κB and can block cell death induced by a variety of agents [69,70]. In the present study we could indeed demonstrate the synergistic effects of naturally occurring polyphenolic compounds resveratrol and curcumin on NF-κB activation and the regulation of expression of its target gene products.

It is not clear whether these effects of resveratrol and curcumin are mediated only by targeting NF-κB; on the contrary, it is absolutely possible that resveratrol and curcumin mediate their effects by targeting more than one cell signalling pathway [71]. However, if this was the case, then the beneficial effects that resveratrol and curcumin might have in OA and RA therapy would be further emphasized, as recent reports have demonstrated that multi-targeted therapy has a better chance of success against inflammation and cancer compared with therapies that aim for a single target [72,73].

It is well known that the cartilage-specific transcription factor Sox-9 is required for expression of cartilage-specific ECM genes [74-76]. We also observed a reduction in collagen type II and Sox-9 expression in chondrocytes after treatment with IL-1β, consistent with previous reports of articular chondrocytes from our and other laboratories [77]. We further observed, however, an inhibition of IL-1β-induced downregulation of collagen type II and Sox-9 expression by pre-treating the cells with resveratrol or curcumin. Moreover, we extended these studies by pre-treating the cells with a combination of resveratrol and curcumin, revealing further inhibition of IL-1β-induced reduction in collagen type II and Sox-9 expression compared with each compound alone. These changes in expression of collagen type II and Sox-9 in response to resveratrol and curcumin are probably due to alternate modes of regulation, independent of changes in NF-κB. Consistent with these findings, previous studies from other laboratories have shown that cytokines partially reduce Sox-9 protein levels over a period of 8 hours through a NF-κB-dependent, post-transcriptional mechanism in mouse chondrocytes [78,79] - demonstrating how active transcription factors, sharing common co-factors, regulate gene expression.

## Conclusions

**Figure 12:**
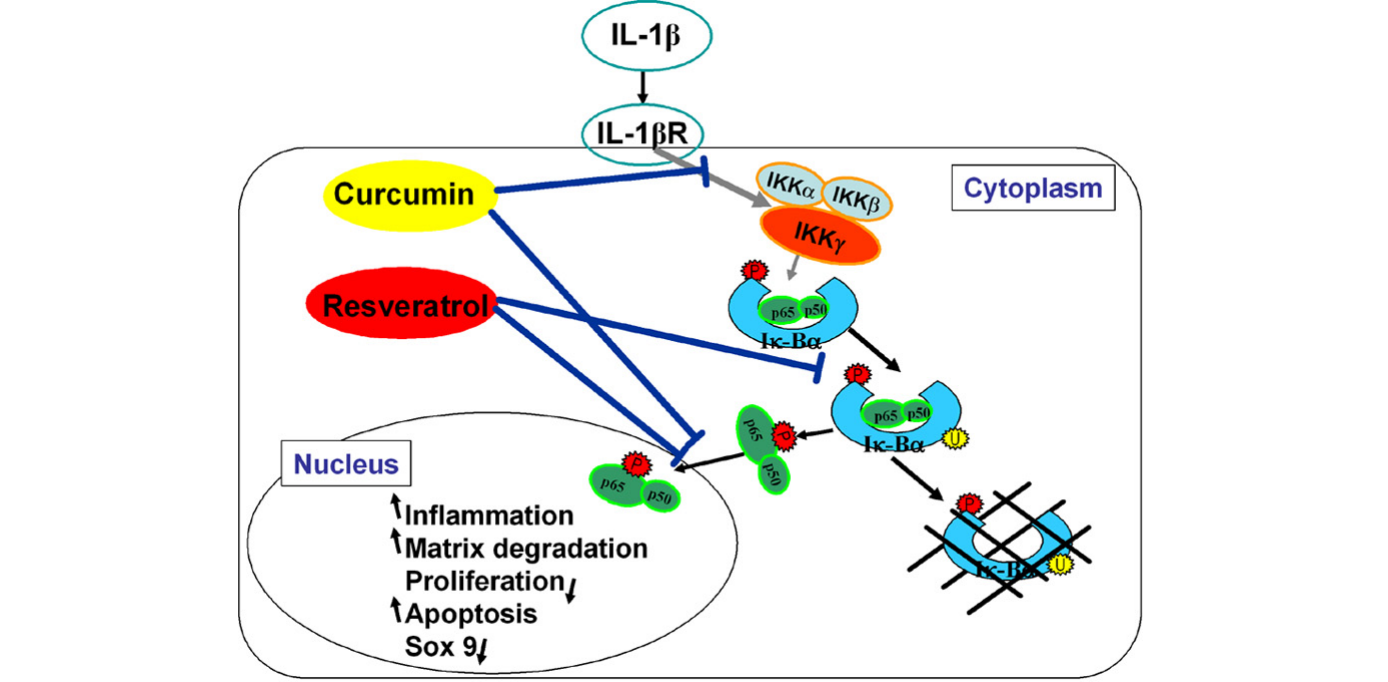
Inhibitory effects of resveratrol and curcumin on IL-1β-induced NF-κB activation and apoptosis in primary human chondrocytes *in vitro*. IL-1β stimulates the IL-1β receptor, intiating an intracellular signal transduction cascade, which activates the cytoplasmic IκBα kinases (IKK)-α, IKK-β, and IKK-γ. These kinases phosphoryle inactive IκBα. Phosphorylated IκBα is then ubiquitinated and degraded by the proteasome and active NF-κB is released. NF-κB translocates to the nucleus, where it activates proinflammatory and pro-apoptotic gene production. In chondrocytes, resveratrol and curcumin both inhibit the NF-κB signal transduction pathway but in different ways: resveratrol inhibits ubiquitination of phosphorylated IκBα, and blocks translocation of the activated NF-κB to the nucleus. In a similar fashion to resveratrol, curcumin also inhibits translocation of the activated NF-κB to the nucleus. However in contrast to resveratrol, curcumin does not have a degradation inhibiting effect on phosphorylated IκBα. Instead, curcumin inhibits IL-1β signalling at an earlier point by inhibition of IKK-α, IKK-β, and IKK-γ.

The results presented here suggest that the anti-inflammatory and anti-apoptotic effects of resveratrol and/or curcumin are mediated through crosstalk among the inhibition of the IKK-induced and proteasome-induced NF-κB pathway, which are activated by a wide variety of proinflammatory agents. Based on these results, we conclude that both resveratrol and curcumin are direct inhibitors of IKK and the proteasome; through this inhibition they block NF-κB and NF-κB-regulated gene expression (Figure 12). However, since a large variety of intracellular signalling pathways interact and converge in chondrocytes, we do not exclude that both resveratrol and curcumin may have additional molecular targets in these cells. Consequently they may also influence inflammatory and apoptotic pathways using other mechanisms.

Further *in vitro *and *in vivo *studies in animals and humans will be required to determine the full potential of the synergistic effects of both resveratrol and curcumin and their potential for the prevention and treatment of OA and RA.

## Abbreviations

ALLN: *N*-Ac-Leu-Leu-norleucinal; APAAP: alkaline phosphatase anti-alkaline phosphatase; BSA: bovine serum albumin; Cox-2: cyclooxygenase-2; DMEM: Dulbecco's modified Eagle's medium; ECM: extracellular matrix; FCS: foetal calf serum; IKK: IκB kinase; IL: interleukin; MMP: matrix metalloproteinase; MTT: 3-(4,5-dimethylthiazol-2-yl)-2,5-diphenyltetrazolium bromide; NF: nuclear factor; OA: osteoarthritis; PARP: poly(ADP-Ribose) polymerase; PBS: phosphate-buffered saline; RA: rheumatoid arthritis; TNF-α: tumour necrosis factor α; TRAF1: TNF-α receptor-associated factor 1; VEGF: vascular endothelial growth factor.

## Competing interests

The authors declare that they have no competing interests.

## Authors' contributions

CC carried out the experimental work, the data collection and interpretation, and the manuscript preparation. AM and MS conceived of the study design, and coordinated the studies, data interpretation and manuscript preparation. All authors read and approved the final manuscript.
